# Increased heart rate after exercise facilitates the processing of fearful but not disgusted faces

**DOI:** 10.1038/s41598-017-18761-5

**Published:** 2018-01-10

**Authors:** G. Pezzulo, P. Iodice, L. Barca, P. Chausse, S. Monceau, M. Mermillod

**Affiliations:** 1 Institute of Cognitive Sciences and Technologies, CNR, Via S. Martino della Battaglia, 44, 00185 Rome, Italy; 2 Université Clermont Auvergne, CNRS, LAPSCO, F-63000 Clermont–Ferrand, France; 3 Université Grenoble Alpes, LPNC, F-38040, Grenoble & CNRS, LPNC UMR 5105, F-38040 Grenoble, France; 4 Institut Universitaire de France, Paris, France

## Abstract

Embodied theories of emotion assume that emotional processing is grounded in bodily and affective processes. Accordingly, the perception of an emotion re-enacts congruent sensory and affective states; and conversely, bodily states congruent with a specific emotion facilitate emotional processing. This study tests whether the ability to process facial expressions (faces having a neutral expression, expressing fear, or disgust) can be influenced by making the participants’ body state congruent with the expressed emotion (e.g., high heart rate in the case of faces expressing fear). We designed a task requiring participants to categorize pictures of male and female faces that either had a neutral expression (neutral), or expressed emotions whose linkage with high heart rate is strong (fear) or significantly weaker or absent (disgust). Critically, participants were tested in two conditions: with experimentally induced high heart rate (Exercise) and with normal heart rate (Normal). Participants processed fearful faces (but not disgusted or neutral faces) faster when they were in the Exercise condition than in the Normal condition. These results support the idea that an emotionally congruent body state facilitates the automatic processing of emotionally-charged stimuli and this effect is emotion-specific rather than due to generic factors such as arousal.

## Introduction

Embodied theories of emotion propose that emotional processing is grounded in *situated conceptualizations* and the re-enactment of the same affective, sensory and motor states that are activated when the emotion is experienced^1–3^. In this perspective, shared brain and bodily resources support the feeling of an emotion, its display, the ability to perceive the emotion when displayed by others, and to perform other related tasks such as processing emotional language, images or faces. Support for this view comes, for example, by evidence showing that the perception of emotion involves areas for feeling the same emotions^4^. This and other studies show that a key role in emotional processing is played by neuronal structures that receive interoceptive feedback on the physiological condition of the body such as the insular cortex, which is considered a key locus of subjective feelings^5–10^ and which is important for adaptive regulation of energy and allostasis^11^.

However, all these studies measure central nervous representations of body state, not physiological aspects of body state. It is less clear which role the body itself plays in emotional processing. More than one century ago, William James argued that physiological changes are able to cause specific emotions rather than being their consequences^12,13^. The so-called *James-Lange hypothesis* has generated controversy and has been re-proposed and extended multiple times. For example, Damasio & Carvalho^14^ suggested that “changes in body state cause automatic physiological reactions as well as mental experiences — feelings — such as hunger, thirst, pain or fear” (p. 143). Along similar lines, Craig^15^ suggested that changes in body state trigger an interoceptive process that gives rise to feelings and emotions in cortical brain areas such as the insula. This theory offers an integrative perspective on emotional processing, which emphasizes a circular causality between somatic/physiological processes and central representations of these processes.

This latter theoretical perspective links well to anatomical evidence that interoceptive afferent fibers monitor the state of all body organs, and report interoceptive signals to cortical structures, thus producing a central representation of homeostatic state and the physiological condition of body tissues, including behaviourally relevant parameters such as bodily temperature and pain^5,15,16^. Importantly, this process culminates in the insula, which is in an excellent position to integrate multimodal information about motivationally salient, hedonic and cognitive/social signals from other parts of the brain, and to use this multimodal information to exert control over the autonomic system it monitors (aka allostasis^11^). In this framework, emotional experiences and feeling states would be associated to the functioning of the cortical system that monitors (and controls) the physiological and homeostatic condition of the body, and in particular to the anterior insula^5^. Support for this idea comes from studies that imply the insula in the processing and awareness of various emotions and feelings^9,17–19^. Hence, in this framework, emotional processing - e.g., feeling an emotion, perceiving or recognizing an emotion - would be inextricably linked (and permeable to) bodily signals as monitored by interoceptive streams.

The aforementioned perspective on allostasis and emotional processing has been recently cast in more formal terms, by appealing to the idea that the brain uses interoceptive signals to implement a form of *interoceptive* or *embodied inference*, in the same way it uses sensory signals to implement perceptual inference^20–24^. Interoceptive inference exploits bottom-up (afferent) and top-down (efferent) interoceptive dynamics in order to maintain a central estimate of physiological and homeostatic parameters of the body, and to take corrective actions (e.g., trigger autonomic reflexes) to control these parameters if they are, or are expected to be, outside safe ranges - thus complying to the overall imperative of minimizing prediction error (or free energy), which is crucial for the survival of the organism^25–27^. Importantly, the internal estimate of bodily parameters formed during this process (and culminating in the insula) would also constitute part and parcel of emotional experience. This perspective is thus coherent with the idea that emotional experience and feelings derive from central representations of the physiological condition of the body and of changes in bodily state^6^. If this hypothesis holds, manipulating interoceptive signals by changing body state should influence emotional processing. Furthermore, this change should be specific; for example, inducing a body state that is congruent with a given emotion (e.g., high heart rate for fear) should facilitate the processing of that emotion, but not of other emotions that are not (or significantly less strongly) associated to the same bodily states (e.g., disgust).

The aim of this study is testing if the ability to process other’s facial expressions (faces expressing fear vs. disgust or neutral faces) can be influenced by manipulating the participants’ heart rate to make it “emotionally congruent” with the observed facial expression (i.e., higher heart rate for faces expressing fear vs. disgust or neutral faces). In keeping with the view that emotional processing is an embodied process, we hypothesize that participants with (experimentally manipulated) increased heart rate are facilitated in processing faces expressing congruent physiological/emotional state (in our study, fear, which is physiologically associated with high heart rate). Comparing one emotion that is strongly associated with high heart rate (fear) and one emotion that is weakly or not associated with it (disgust) will permit us to assess whether a facilitatory effect is specific to a body state (and its associated interoceptive signals) or is due to a more generic process that results from physical activity such as arousal - intended here as a generic state of physical/mental alertness and readiness to move or to process stimuli, which is not univocally associated to a particular emotion^28^.

To test whether our “embodiment of fear” hypothesis holds, we designed an experiment in which participants were tested into two conditions: an *Exercise condition* in which they performed physical exercise before the experiment, causing an acceleration of heart rate; and a *Normal condition*, in which they performed no physical exercise before the experiment. Participants were presented with pictures of male and female faces with *fear*, *disgust* or *neutral* expressions and performed a gender categorization task. Our central hypothesis was that high heart rate (induced by physical exercise) would have a facilitatory effect on the processing of fearful faces (because fear is congruent with high heart rate) but not of disgusted or neutral faces.

We follow a consolidated tradition, especially in the human neuroimaging literature, of studying emotional processing by using *incidental* tasks - and especially gender categorization tasks - whose demands are orthogonal to the real goals of the experiment, rather than by asking participants to process emotional content explicitly^29–31^, in order to avoid confounds with other (e.g., metacognitive) processes or methodological problems such as demand biases^32^. Studies comparing direct and incidental processing of emotional content have validated this methodology by showing that both tap emotion-related brain circuits^33–35^. The argument underlying our incidental task is that, if high heart rate increases the sensitivity to fear-related stimuli, it will influence both emotional and non-emotional judgments. Measuring participants’ speed and uncertainty during gender categorization would thus offer an unbiased window over our variable of interest: namely, the congruency between their body state and different emotions displayed during the incidental task. In other words, using an incidental task would provide a strong proof of automatic influences of physiological state on emotional processing (and *excitation transfer* ^36^) that are unconfounded by other, more explicit (e.g., metacognitive) processes or explicit attention regulation^37^.

Subjects provided their responses by moving a computer mouse on the selected category. Recent studies using mouse movements (or other continuous kinematic measures) permitted to shed light into the dynamic properties of the moment-to-moment decision process and have been applied to a number of paradigms, such as numerical and color comparisons, categorization of ambiguous figures, and semantic categorization^38–46^. Similarly, in this study we collected two kinematic measures in order to reveal the online dynamics of the decision process, and in particular how the choice uncertainty is reflected in the trajectory curvature^47,48^. In particular, we focused on two kinematic measures: Maximum Deviation (MD) and Area Under the Curve (AUC). MD is the length of a perpendicular line between the idealized straight-line trajectory and the farthest point from that straight-line in the observed trajectory. AUC is the geometric area between the observed mouse-trajectory and an idealized straight-line trajectory drawn from the start and end points. Both measures assess the degree of attraction toward an unselected response^49^. We expect faster responses and more direct mouse trajectories (assessed by MD and/or AUC) when the presented stimulus was congruent with the body state of the participant (e.g. fearful faces after exercise) compared to incongruent situation (e.g. neutral faces or with expression of disgust after exercise).

## Methods

A group of 24 male students of the G. D’Annunzio University of Chieti (Italy) with ages ranged from 20 to 26 years were recruited for participation. Participants were members of the same cultural group (i.e., Caucasian). All were right-handed with normal or corrected to normal vision. Our sample was composed only by male subjects to avoid confounds due to gender related factors (for example, many studies have found women to be more emotionally responsive than men, particularly when processing facial expressions^50–52^). Informed consent was obtained from each participant and the study protocol conforms to the ethical guidelines of the Declaration of Helsinki (BMJ 1991; 302; 1194) as reflected in prior approval by the Institution’s human research committee (ISTC-CNR, Rome - N.0003971/04/12/2015).

Experimental stimuli comprised 120 pictures of male or female faces, varied for facial expression (neutral, disgust and fear). We used 60 male faces (20 neutral, 20 fearful, 20 disgust emotional expressions) and 60 female faces (20 neutral, 20 fearful, 20 disgust emotional expressions). The same facial identities were used to depict each of the three expressions. The stimuli used came from the Karolinska Directed Emotional Faces (KDEF) database. Color images were transformed to 256 gray-level scale. We then normalized the luminance of the images and applied a Hann window in order to remove the hair and the peripheral information of the faces^53–55^. This was done in order to avoid a categorization of the gender based on the hair of the faces^56^. Different combinations of facial expression produce the following categories: female neutral, female fear, female disgust, male neutral, male fear, and male disgust.

On each of the 120 trials, participants clicked on the/START/button located at the bottom-center of the PC screen. Then a face appeared centrally and, in order to perform the gender categorization task, participants were asked to click on the ‘male’ or ‘female’ response in the top-left and top-right corner of the screen. Rightward and leftward responses were counterbalanced across participants. A time deadline of 1400 ms was used, and responses exceeding it resulted in the appearance of a ‘time out’ message. Trials were presented in two blocks with a pause in between. A practice session of 8 trials familiarized the participants with the procedure. During participants’ responses, the x and y coordinates of the mouse trajectories were recorded (sampling rate of approximately 70 Hz) using the MouseTracker software^47^.

All participants (n. 24) performed the experiment in two conditions: in the *Exercise condition* they were asked to perform physical exercise (i.e., stepper) for 3 minutes, at their own rhythm, before the actual experiment, and during the pause between the experimental blocks. This procedure permitted to control that the heartbeat of participants of the *Exercise condition* did not come down to a normal rhythm during the execution of the experimental task (see below). In the *Normal condition*, participants performed the experiment without any preceding exercise. Rather, before the experiment and after each experimental block, they were asked to relax on their chair for 3 minutes (in order to make the timing of two conditions as similar as possible). The order of conditions was counterbalanced: 12 subjects performed the experiment in the *Exercise condition* first, and in the *Normal condition* one week later, at approximately the same hour of the day; the other 12 subjects followed the opposite order. The position of the two response buttons (male and female) was counterbalanced, too.

During the experiment, a Polar RS800CX (which records HR beat by beat at +/−1 ms) was used to record variations in participants’ Heart Rate (HR). The experimenter placed new ECG electrodes to measure heart rate before and during each block of the experiment. Participants in the Exercise condition have average heart rate of 118 ± 8 bpm during the experiment. Participants in the Normal condition have average rate of 71 ± 8 bpm.

The dependent variables were participants’ overall response time (RT), two kinematic parameters of movement (velocity and acceleration peaks) and three parameters that measure movement trajectories during the response: Area Under the Curve (AUC) and Maximum Deviation (MD) of the trajectories. Following standard procedures used in MouseTracker studies, response trajectories were first rescaled into a standard coordinate space, and the duration of the movements were normalized by re-sampling the time vector into 101 time-steps using linear interpolation to allow averaging across multiple trials^47^. Responses exceeding the time deadline (0.47% of the total data), with standard deviations (SD) greater than 3 times average of reaction time (5.4%) and incorrect categorization (11% of trials) were discarded from analysis.

Linear mixed-effects models (LMMs)^57^ were used to analysed response time data, with the ‘lmerTest’ package^58^. Backward elimination of non-significant effects was performed with the step function (backward elimination of the random part is performed first, followed by backward elimination of the fixed part. Finally, least squares means and their differences for the fixed part of the model are calculated. The p-values for the fixed effects are calculated from an F test and t test based on Sattethwaite’s approximation). The model included random intercept for Subjects and Items, with maximal by-subject random structure as a baseline model, and Facial Expression (Neutral, Disgust, Fear) and Session (Exercise, Normal) as fixed effects.

## Results

**Figure 1:**
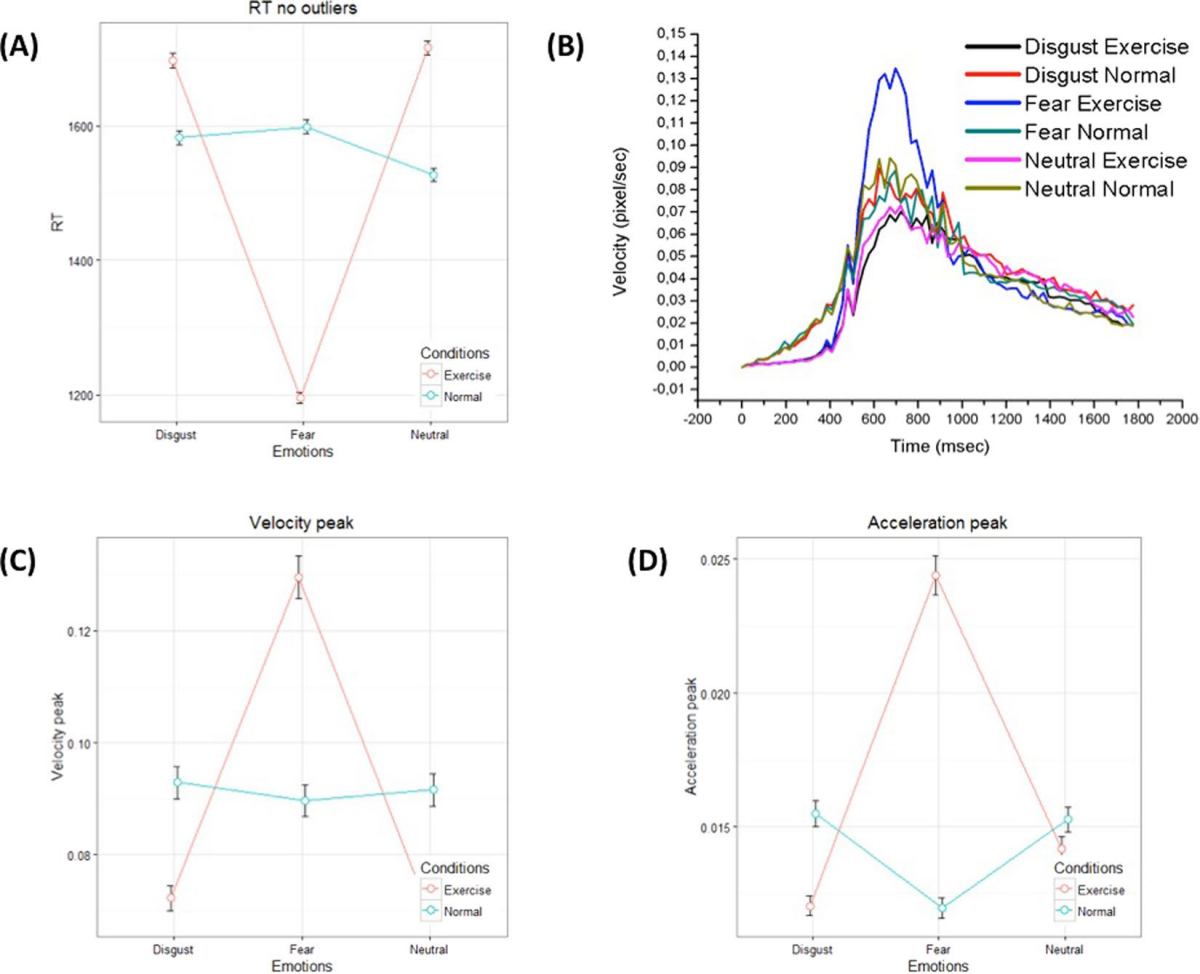
Mouse tracking kinematic data. (**A**) Correct response time as a function of Session (Exercise, Normal) by Facial Expression (Neutral, Disgust, Fear). Error bars depict Standard Error of the Mean. (**B**) X-velocity profile of the mouse movements of participants processing faces with disgust (exercise, black; rest orange), fear (exercise, violet; rest, green) and neutral (exercise, light violet; rest, ochre) expressions. (**C,D**) Velocity peaks (**C**) and acceleration peaks (**D**) of mouse movements as a function of Session (Exercise, Normal) by Facial Expression (Neutral, Disgust, Fear). Peaks are reported in Table 1.

About 3.7% of total data points were removed as response outliers (identified with the Turkey’s method, which identifies the outliers ranged above and below the 1.5* interquartile range of response time). The analysis of correct response time showed a significant main effect of Facial Expression (F _(2, 22.12)_ = 213.3, p-value < 0.001), and a significant Facial Expression by Session interaction (F _(2, 8229.59)_ = 783.41, p-value < 0.001); see Fig. 1A. The main effect of Session was kept in the model but was not significant (F _(1, 22.93)_ < 1, ns). A significant difference emerged between Fear Exercise and Fear Neutral (estimate of differences in LSMEANS = −408.2, SE = 41.7, DF = 25.6, t-value = −8.76, p-value < 0.001), with faster response to fearful faces from the Exercise group. Differences among groups emerged also for neutral and disgust-related faces, but in the opposite direction, that is, slower responses for the Exercise group (estimate of differences in LSMEANS_Neutral_ = 206.6, SE = 41.7, DF = 25.7, t-value = −1.09, p-value < 0.001; estimate of differences in LSMEANS_Disgust_ = 132.6, SE = 41.8, DF = 25.8, t-value = 3.18, p-value < 0.005, respectively).

**Table 1:** Velocity and acceleration peaks. Velocity peak is calculated as the maximal value in the velocity profile, in the x-axis. Acceleration peak is calculated as the maximal value in the acceleration profile. Peak latencies were defined as the time elapsed between movement onset and maximum peak amplitude.

	Velocity Peak		Acceleration Peak	
	Time (ms)	Mean amplitude (pixel/sec)	Time (ms)	Mean amplitude (pixel/sec)
Neutral rest	672	0,09414	625–648	0,01675
Neutral Exercise	720	0,07301	553–576	0,01464
Fear Rest	696	0,08862	553–576	0,01174
Fear Exercise	696	0,13451	553–576	0,02442
Disgust Rest	672	0,08962	625–648	0,01884
Disgust Exercise	720	0,07014	553–576	0,01085

The analysis of velocity and acceleration peaks in the x-axis during the movement (see Table 1 and Fig. 1) shows almost the same pattern as reaction time. Analysis of velocity peak data showed a significant main effect of Facial Expression (F_(2, 8528.93)_ = 75.29, p-value < 0.001) and the interaction Session by Facial Expression (F_(2, 8469.2)_ = 89.51, p-value < 0.001); see Fig. 1B,C. Main effect of Session was not significant (F_(1, 23)_ < 1, ns). A significant difference emerged between Fear Exercise and Fear Neutral (SE = 0.014, DF = 25.3, t-value = 2.88, p-value < 0.05), with a higher peak of velocity in the response to fearful faces from the Exercise group. No other differences were significant (SE_Neutral_ = 0.014 DF = 25.3, t-value = −1.5, ns; = 132.6; SE_Disgust_ = 0.014, DF = 25.3, t-value = −1.48, ns).

The analysis of acceleration peak showed significant effect of Facial Expression (F_(2,8588.7)_ = 53.29, p-value < 0.001) and interaction (F_(1,8588.7)_ = 181.53, p-value < 0.001); the main effect of Session was not significant (F_(1,23)_ = 1.37, ns); see Fig. 1D. Like in the analysis of velocity peak, the analysis reveals a higher acceleration in the Exercise session with fearful faces compared to neutral faces (SE = 0.0006, DF = 25.5, t-value = 5.38, p-value < 0.001). No differences emerged with disgust-related faces (SE = 0.0023, DF = 25.5, t-value = −1.49, ns) or neutral faces (SE = 0.0023, DF = 25.5, t-value = −0.47, ns).

**Figure 2:**
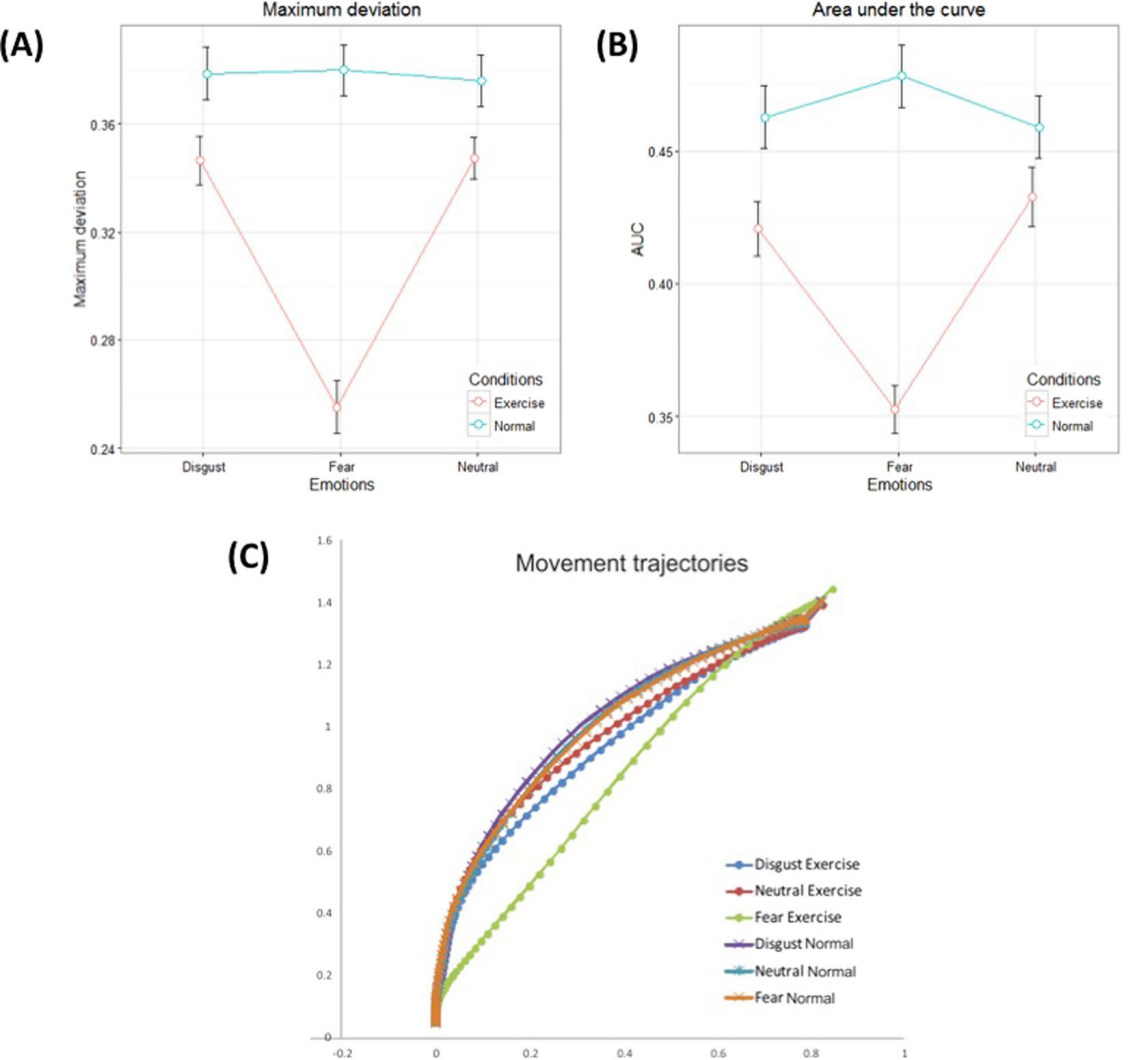
Mouse tracking trajectory data. (**A**) Maximum deviation MD and (**B**) Area Under the Curve AUC as a function of Session (Exercise vs. Normal) by Facial Expression (Neutral, Disgust, Fear) interaction. Error bars depict Standard Error of the Mean. (**C**) Average spatial trajectories of the responses of disgust (Exercise, blue; Normal, violet), neutral (Exercise, red; Normal, light blue) and fear (Exercise, green; Normal, orange).

On maximum deviation (MD), we found a significant main effect of Session (F_(1,23)_ = 9.47, p-value < 0.05), Facial Expression (F_(2,8572.17)_ = 15.54, p-value < 0.001), and their interaction (F_(2,8539)_ = 18.565, p-value < 0.001). Significant differences of LSMEANS emerged between Exercise vs. Normal sessions with fear-related faces (SE = 0.0226, DF = 37, t-value = −5.54, p-value < 0.001). No differences emerged with disgust-related faces (SE = 0.0226, DF = 37.1, t-value = −1.41, ns) or neutral faces (SE = 0.0226, DF = 37, t-value = −1.25, ns), see Fig. 2A.

A similar patter of results emerged on the measure of area under the curve (AUC), with significant fixed-effect of Session (F_(1,8431.98)_ = 56.93, p-value < 0.001), Facial Expression (F_(2,8600.56)_ = 3.72, p-value < 0.05) and interaction (F_(2,8601.27)_ = 12.48, p-value < 0.001). Significant differences of LSMEANS emerged between Exercise vs. Normal sessions in fear-related faces (SE = 0.0150, DF = 8582.3, t-value = −8.37, p-value < 0.001), and to a lesser extent, disgust-related faces (SE = 0.0150, DF = 8588.3, t-value = −2.77, p-value < 0.05). No differences emerged with neutral faces (SE = 0.0149, DF = 8556.5, t-value = −1.81, ns); see Fig. 2B. See also Fig. 2C for an illustration of average mouse trajectories during the task.

We conducted an additional analysis to assess if the effects we found were gender-specific, i.e., specific for male versus female faces. In the analysis of reaction time, there was no significant effect of gender (F_(1, 24.23)_ < 1, ns), no interaction condition by gender (F_(1,8160.8)_ < 1, ns) or facial expression by gender (F_(2,8153)_ < 1, ns). The same pattern emerged also for peak velocity (Gender: F_(1, 8532.21)_ = 1.23, ns; Condition by Gender: F_(1,8564.82)_ < 1, ns; Facial Expression by Gender: F_(2,8542.54)_ = 1.45, ns) and peak acceleration (Gender: F_(1, 8582.7)_ = 1.33, ns; Condition by Gender: F_(1,8582.7)_ < 1, ns; Facial Expression by Gender: F_(2,8582.7)_ = 1.86, ns).

## Discussion

Our results indicate that participants whose heart rate was experimentally manipulated (raised) recognized faster faces expressing fear - an emotion that is strongly congruent with high heart rate - compared to faces expressing disgust - an emotion having significantly weaker linkage (or no linkage) with high heart rate - or neutral faces. This conclusion is supported by convergent evidence obtained by measures of reaction time and peak velocity and acceleration during the response. Furthermore, two measures of choice uncertainty during the choice, maximum deviation (MD) and area under the curve (AUC), support the conclusion that participants whose heart rate is higher are not only faster in recognizing faces expressing fear compared to a control group, but also less uncertain during the choice. In sum, these results indicate that a rather mundane manipulation of physiological and interoceptive state is sufficient to produce an “embodied congruency” effect between body and interoceptive state (high heart rate) and stimuli charged with congruent emotional content (faces expressing fear).

Importantly, the facilitation in perceptual processing was only evident for faces expressing fear, but not faces expressing disgust or neutral faces, highlighting the specificity of the effect. Reassuringly, our results are coherent across different measures: reaction time, kinematic measures of peak velocity and acceleration, and measures of maximum spatial deviation from an ideal straight trajectory and area under the curve of this deviation – where the two latter measures are associated with choice uncertainty. This coherent pattern of results indicates that the facilitating effect of high heart rate depends on its congruency with the emotion of fear, not on more generic factors (e.g., a generic emotional or arousal state that may facilitate the recognition of all emotional stimuli, or speed up overall response time independent of the stimuli). This study thus replicates and extends previous findings that facial expressions and body arousal can influence the recognition of emotionally charged stimuli^28,59–64^, highlighting the importance of congruent bodily states. A further indication of that the Exercise condition did not produce a generic facilitatory effect comes from the fact that participants in this condition were generally slower (not faster) in recognizing disgusted and neutral faces. At the same time, the Exercise condition determined trajectories that deviated less from an ideal trajectory - although this effect was only significant for fearful faces in the case of maximum deviation, and more pronounced for fearful compared to disgusted faces in the case of area under the curve (see also Fig. 2C). A careful analysis of the velocity profiles of the spatial trajectories (Fig. 1B) helps understanding this pattern of results. As shown in Fig. 1B, mouse movements were initially slower in the Exercise condition, which would in general increase reaction time but produce less choice uncertainty^65^. A slower initial phase of movement would thus explain why we observed both slower reaction times and decreased choice uncertainty in the Exercise condition. Figure 1B shows that when participants in the Exercise condition processed fearful faces, their velocity peak was higher - which explains their faster reaction time. Interestingly, in this case latter decrease in uncertainty goes hand in hand with faster (not slower) reaction time, which is the hallmark of a facilitatory effect.

The fact that the processing of fearful faces is only facilitated in the Exercise (but not the Normal) condition rules out the possibility that fearful faces where easier to discriminate than disgusted or neutral faces. This comparison allows us to rule out other possible alternative explanations, too, such as the possibility that showing people fearful faces could in itself increase heart rate, which in turn could enhance their processing. If that was the case, we should have observed faster processing of fearful faces in both Exercise and Normal conditions; but we do not. This pattern of results thus indicates that the facilitatory effect was due to congruence between emotional stimuli and experimentally-manipulated bodily state, not to the mere presence of an emotionally-charged stimulus (e.g. a fearful face).

Finally, the fact that the facilitation was present in an incidental cognitive task (a gender categorization task) that did not require subjects to evaluate explicitly the emotional content of the stimuli suggests that it rests on low-level inferential mechanisms rather than higher (e.g., metacognitive) processing stages of emotional content - which is in keeping with the results of other recent experiments^29–31^. Our study adds to this body of work by shedding light on the possible interactions between physiological state and the processing of emotional stimuli. A widespread debate concerns the “direction of causality” between emotional and bodily processes: does the emotion of fear elicit high heart rate, or does high heart rate elicit emotion - or both? The idea that changes in bodily state are part and parcel of emotional processing can be traced back at least to the *James-Lange hypothesis* ^12,13^ and is key to many modern theories of embodied emotion and interoception^14,15,66^. One central claim of this approach is that changes in body state can produce emotional states and thus plausibly influence emotional processing at large. Our results support this view, by showing that the modification of the physiological state of the participants’ bodies (increasing heart rate) facilitated the implicit processing of congruent stimuli (faces expressing fear) but not of other emotional (disgust) or neutral stimuli. While changes in arousal measures during emotional processing have been extensively reported^67^ here we report the reverse relation: manipulating the subjects’ body state can influence emotional processing, even in an *incidental task*.

The empirical basis for an association of fear and high heart rate is large, and several studies report unchanged or even lowered heart rate during the processing of disgusting stimuli^68–71^. However, a case has been made also for a linkage between disgust and increased heart rate under specific circumstances^72^. Specifically, the literature distinguishes between two disgust-related body states - core disgust (related to, e.g., food contamination) and body-boundary violation (BBV) disgust (related to, e.g., mutilation and blood) - and a case has been made that HR may remain stable or sometimes increase in the former (core disgust) case and decrease in the latter (BBV) case^72^. Interestingly, core and BBV disgust also correspond to partially different facial expressions^73^. More than 63% (38 out of 60) of the stimuli in our study show the most characteristic features of faces expressing BBV disgust (upper lip retraction), while no stimuli in our study show the two most characteristic features of faces expressing core disgust (mouth gape or tongue protrusion)^73^. According to the taxonomy of ^73^, our stimuli thus largely fall within the sub-category of facial expressions related to BBV disgust, for which there is no potential confound with high heart rate. Finally, and most importantly in this context, there is ample evidence from studies comparing fear and disgust that heart rate is significantly higher in the former case, hence providing a rationale for our comparison^71,74,75^. In other words, even if one places fear- and disgust-related stimuli along a continuum (in relation to high heart rate), our experimental manipulation would be expected to affect the processing of fear-related stimuli significantly more than disgust-related stimuli - which is exactly what we report.

The findings reported in this paper may be also explained by assuming that energy expenditure (i.e., exercise) leads to quicker processing of facial expressions related to resource and behavioral mobilization. Since exercising is an act of resource expenditure and mobilization (similar to “running away”), perceivers who performed an exercise very recently may be able to more quickly perceive facial expressions of fear, which (compared to disgust or neutral expressions) signal a threat in the environment and the necessity of bodily action. This explanation is not incoherent with our arguments, if one considers that the plausible evolutionary basis for high heart rate in fear processing is exactly the fast mobilization of resources in case of threat; and that the embodiment of fear plausibly encompasses a general state of bodily resources mobilizations, not just heart rate acceleration. In other words, while this alternative explanation highlights the link between energy expenditure (i.e., exercise) and fear processing rather than between high heart rate and fear processing, all these phenomena are tightly linked at the physiological level (e.g., exercise causes high heart rate) and from an adaptive viewpoint.

This study adds to a large body of literature on emotional processing and its embodiment. This body of literature is sometimes fragmented or even contradictory; indeed, despite its pervasiveness in human life, emotion has revealed to be an elusive phenomenon to define and to assess empirically^14,76^. One recurrent question in this literature is whether it is possible to find emotion-specific patterns in peripheral autonomic and central neural responses^77,78^. A meta-analysis of neuroimaging studies indicates that consistent neural correlates of specific emotions (e.g., happiness and sadness) can be found across studies^79^. Furthermore, a recent study highlights that it is possible to clearly dissociate two disgust forms by looking at their distinct patterns of autonomic and central responses^66^; and another study reports that fearful faces were detected more easily and were rated as more intense when the timing of their presentation was congruent to the subjects’ individual heartbeats^80^. At the same time, there have been some failed attempts to induce specific emotional states via electrical stimulation^81^, and this has lead to the suggestion that several other dimensions such as social aspects should be considered to fully understand emotional processing, besides primary emotion circuits. Embodied theories of cognition have the potential to play an integrative role, by suggesting that emotion is a multifarious phenomenon and it links to a multitude of situational, affective, perceptual and bodily states, all of which play a role in emotional processing^1–3^. In this perspective, emotional processing can be conceptualized as an embodied simulation that elicits modal circuits for sensory and affective states that are active whenever an emotion is experienced - consistent with the general claim of embodied theories that cognition is based on embodied simulation and the re-enactment of modal brain circuits^3,82–87^.

This claim has been addressed by studies that manipulated proprioceptive and motor circuits (e.g., face muscles) to make them more or less congruent with emotional stimuli (e.g., faces expressing emotions) and which showed an influence of peripheral events (subjects’ facial expressions) on emotional processing^59–64,88,89^. At difference with those studies, which focused on muscular movements and motor (or proprioceptive) channels, here we targeted interoceptive channels - which are increasingly recognized to be key to emotional experience, feeling, and consciousness^8,14,20,22,23,90^. Our study thus constitutes a more direct test of the idea that bodily state influences emotional processing through interoceptive channels. A possible explanation of the relations between bodily state and the perception of emotional content is offered by theories of *emotion resonance* ^91^. In this perspective, feeling an emotion can influence emotion perception via direct, resonant mechanisms in the brain, much like performing an action influences action perception^92,93^. An alternative hypothesis stems from recent theories of *interoceptive predictive coding* ^22,27,94^, *embodied predictive coding* ^21,26,95^ and *Embodied Predictive Interoception Coding (EPIC)* ^20,96^, all of which describe the processing of interoceptive events, or of combined perceptual and interoceptive events, in terms of surprise (or free energy) minimization^25^. For example, the theory of embodied predictive coding^21^ suggests that perceptual inference combines sensory and interoceptive streams of evidence - more specifically, it tries to simultaneously minimize prediction errors between hypotheses and sensations in both streams - and so it is considerably faster when the emotional state of the participant is congruent with that of the to-be-recognized figure (as the prediction error to be minimized is smaller). The error-minimization mechanism implicit in predictive coding and interoceptive inference is analogous to a *situated simulation* of emotionally charged events, when the current bodily state is considered as part of the overall experience to be re-enacted during perceptual processing^3^. The specific hypotheses proposed here remain to be tested empirically; however, they all highlight the importance of fusing information processing streams in “the brain” and “the (rest of the) body” and all assign the body a central stage in perceptual and conceptual processing, as James and Lange hypothesized long time ago.

This result also links to a body of literature showing bidirectional interactions between “emotion” and “cognition”; for example, a recent meta-analysis discusses several studies where emotions (e.g., happiness and sadness) were elicited that caused correlated changes in behavior, experience, and physiology^97^. However, it is less clear what is the adaptive value of these interactions; for example, several so-called “dual theories” challenge that so-called “emotional” (or “hot”) forms of processing can be deleterious for an otherwise supposedly “rational” (or “cold”) cognition^98^. Contrary to the idea that emotional processing makes so-called “rational” behaviour less efficacious, several authors have argued that these bidirectional interactions can have adaptive value^76,99,100^. In this vein, it is possible to speculate that increased arousal and faster emotional processing revealed in our study can be linked to the adaptive value of responding faster to dangerous situations. This idea is in keeping with recent studies showing that also perception is modulated by valence; for example, threatening objects such as spiders appear to move faster^101^ and be closer and larger^102^ than non-threatening objects; see also^103^. Taken together, this body of evidence suggests that both the exteroceptive (sensory) and interoceptive (including affect-related) dimensions are not neutral but tuned to facilitate action and a cost-benefit analysis of the situation^104,105^.

## Conclusion

The ways emotional and perceptual processing interact have always fascinated scholars and laymen, but our current understanding of these problems is still incomplete. In the current experiment, we asked whether inducing a physiological state that was congruent with to-be-recognized emotional stimuli facilitated their processing. Participants in the high-heart-rate group (Exercise condition) showed a faster gender categorization of faces depicting emotional expressions that were congruent with their body state (fearful faces) but not of faces depicting incongruent emotional expressions (disgusted faces) or of neutral faces. In keeping with our main hypothesis, these results suggest that the induction of an emotionally congruent bodily state influences the perception of other’s congruent facial expressions, even in the case of an *incidental* cognitive task. These results thus provide support for embodied theories of emotion, which suggest that bodily processes are part and parcel of emotional processing and not just irrelevant byproducts – or, in other words, that somatic and interoceptive states such as high heart rate participate in the embodiment of fear.
